# Bone Metastasis Mediates Poor Prognosis in Early‐Onset Gastric Cancer: Insights Into Immune Suppression, Coagulopathy, and Inflammation

**DOI:** 10.1002/cam4.70737

**Published:** 2025-03-05

**Authors:** Shi Yin, Xiaohui Zhai, Yaoying Li, Ruixin Zeng, Di Zhang, Xiaoqing Sun, Ziying Zhang, Huashe Wang, Caiqin Wang

**Affiliations:** ^1^ The Affiliated LiHuiLi Hospital of Ningbo University Ningbo Zhejiang China; ^2^ Department of Medical Oncology The Sixth Affiliated Hospital of Sun‐Yat sen University Guangzhou People's Republic of China; ^3^ Guangdong Provincial Key Laboratory of Colorectal and Pelvic Floor Diseases The Sixth Affiliated Hospital, Sun Yat‐sen University Guangzhou People's Republic of China; ^4^ Biomedical Innovation Center The Sixth Affiliated Hospital, Sun Yat‐sen University Guangzhou People's Republic of China; ^5^ State Key Laboratory of Oncology in South China Sun Yat‐Sen University Cancer Center Guangzhou China; ^6^ Department of Medical Oncology, State Key Laboratory of Oncology in South China Guangdong Provincial Clinical Research Center for Cancer, Sun Yat‐sen University Cancer Center Guangzhou P. R. China; ^7^ Ningbo Geriatric Rehabilitation Hospital Ningbo Zhejiang China; ^8^ Department of Intensive Care Medicine (ICU), State Key Laboratory of Oncology in South China, Guangdong Provincial Clinical Research Center for Cancer Sun Yat‐Sen University Cancer Center Guangzhou P.R. China; ^9^ Department of Radiation Oncology, Hunan Cancer Hospital and the Affiliated Cancer Hospital of Xiangya School of Medicine Central South University Changsha Hunan People's Republic of China; ^10^ Department of Gastrointestinal Surgery The Six Affiliated Hospital, Sun Yat‐sen University Guangzhou China; ^11^ Department of Lymphoma and Hematology The Affiliated Cancer Hospital of Xiangya School of Medicine, Central South University, Hunan Cancer Hospital Changsha Hunan China

**Keywords:** bone metastasis, cytokines, early‐onset gastric cancer, mediation analysis, prognosis, survival analysis

## Abstract

**Background:**

The increasing incidence of gastric cancer (GC) in younger populations, coupled with population aging, has highlighted distinct age‐related subtypes with unique clinical characteristics and outcomes. Although younger patients tend to have more aggressive tumors, the prognostic factors for early‐onset gastric cancer (EOGC) remain underexplored. This study is dedicated to providing a comprehensive and in‐depth analysis of prognostic factors in EOGC, aiming to refine personalized treatment strategies under the precision medicine paradigm.

**Methods:**

This retrospective study encompassed 413 local cohort EOGC patients and 8447 Surveillance, Epidemiology, and End Results database patients diagnosed with GC. Survival outcomes were assessed using Kaplan–Meier survival curves, and differences between groups were evaluated with the log‐rank test. Prognostic factors were identified through logistic regression and Cox proportional hazards models. Mediation analysis was conducted to assess the indirect effects of clinical factors on EOGC and prognosis. Biomarker comparisons between bone metastasis early‐onset gastric cancer and non‐bone metastasis early‐onset gastric cancer groups were evaluated using the Wilcoxon test for significant differences.

**Results:**

The overall survival and cancer‐specific survival rates in the EOGC group were significantly lower than those in the non‐early‐onset gastric cancer group (*p* < 0.05). However, EOGC itself was not an independent risk factor for poor prognosis. Mediation analysis revealed that the adverse impact of EOGC on prognosis was predominantly mediated by metastasis, with bone metastasis identified as the most significant factor. Furthermore, bone metastasis emerged as an independent predictor of poor prognosis in EOGC patients, potentially linked to elevated coagulation markers, increased inflammation‐related cytokines, and an imbalance in peripheral blood immune cell ratios.

**Conclusions:**

Bone metastasis significantly contributes to the poor prognosis of EOGC. EOGC patients with bone metastasis demonstrate immune suppression, inflammation activation, and coagulopathy, highlighting the need for tailored management and prognostic strategies.

## Graphical abstract

This study highlights the significant impact of bone metastasis on the prognosis of early‐onset gastric cancer (EOGC). Patients with bone metastasis exhibit a unique pathological profile characterized by immune suppression, inflammatory activation, and coagulation imbalances, creating an environment that promotes tumor progression. Key findings include elevated pro‐inflammatory cytokines and coagulation markers, emphasizing the need for targeted therapeutic strategies to improve outcomes in bone metastatic early‐onset gastric cancer patients.

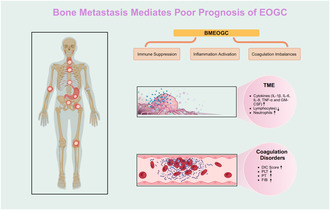

**Abbreviations**
AJCC: American Joint committee on cancer
AKP: alkaline phosphatase
BMEOGC: bone metastasis early‐onset gastric cancer
CI: confidence intervals
CSS: cancer‐specific survival
DIC: disseminated intravascular coagulation
EOGC: early‐onset gastric cancer
FIB: fibrinogen
GC: gastric cancer
HR: hazard ratios
IL‐6: interleukin‐6
LDH: lactate dehydrogenase
LOGC: late‐onset gastric cancer
LY: lymphocyte
nBMEOGC: non‐bone metastasis early‐onset gastric cancer
NE: neutrophil
nEOGC: non‐Early‐onset gastric cancer
OS: overall survival
PLT: platelet count
PT: prothrombin time
RANKL: nuclear factor kappa‐Β ligand
SAH‐SYSU: the sixth affiliated hospital of Sun Yat‐sen university
SEER: surveillance epidemiology and end results
TGF‐β: transforming growth factor‐beta
WBC: white blood cell

## 1 Introduction

Early‐onset gastric cancer (EOGC), typically defined as gastric cancer (GC) occurring in individuals aged ≤ 45 years, has exhibited a steady increase in incidence globally, which signals a concerning trend toward the younger onset of this disease [1, 2]. This rise in EOGC cases underscores the need for greater attention to this distinct clinical subtype. Clinically, EOGC tumors typically manifest with heightened aggressiveness and are frequently diagnosed at advanced stages, characterized by rapid progression and increased metastatic potential [3, 4].

Previous studies typically grouped all patients over 45 into a single category for comparison with EOGC outcomes, often neglecting the substantial survival disparities across different age brackets [5, 6, 7]. This classification method particularly overlooks patients over 75 (who are commonly excluded in clinical studies), whose prognosis is predominantly affected by comorbidities and overall health status, thus confounding age‐related assessments in GC [8, 9]. To enhance our understanding of distinct prognostic factors within EOGC, our approach compares EOGC with a non‐early‐onset gastric cancer (nEOGC) cohort, defined specifically as patients aged 45–75 years. This methodology eliminates confounding factors linked to advanced age, providing a clearer insight into the prognostic implications of EOGC and facilitating a more accurate exploration of its impact [10].

Additionally, studies have shown that EOGC possesses distinctive molecular and biological characteristics that often lead to more aggressive tumor behavior. Specifically, cancer cells create a complex immune microenvironment within the tumor that supports growth and promotes immune suppression and chronic inflammation, all of which contribute to the metastatic propensity of EOGC [11, 12]. Bone metastasis, a critical late‐stage event in EOGC, involves complex interactions within the bone microenvironment, which consists of osteoclasts, osteoblasts, immune cells, and stromal components. Tumor cells disrupt the normal balance of bone remodeling, promoting osteoclastic resorption and osteoblastic formation, thereby creating a niche that facilitates metastasis [13]. This process is further driven by factors such as transforming growth factor‐beta (TGF‐β), interleukin‐6 (IL‐6), and receptor activator of nuclear factor kappa‐Β ligand (RANKL), which not only enhance tumor cell survival and proliferation but also contribute to immune evasion and chronic inflammation [14, 15]. Bone metastasis in EOGC is also marked by coagulation abnormalities, which create a hypercoagulable state aiding tumor cell survival within the circulatory system and providing a scaffold for adhesion and growth [16, 17, 18].

Given these complexities, this study aims to thoroughly assess the impact of age on gastric cancer prognosis and explore the underlying molecular biological mechanisms driving these effects.

## 2 Method

### 2.1 Patient Selection

This study included two independent cohorts of GC patients: the local cohort (*n* = 413) and the Surveillance, Epidemiology, and End Results (SEER) cohort (*n* = 8447). The local cohort consisted of patients diagnosed with EOGC at the Sixth Affiliated Hospital of Sun Yat‐sen University (SAH‐SYSU) between August 2008 and August 2021. Data for the SEER cohort were obtained from cases diagnosed between January 2010 and December 2018. Data extraction was carried out using SEER*Stat version 8.3.6 (http://seer.cancer.gov/seerstat/) (Figure S1).

**FIGURE 1:**
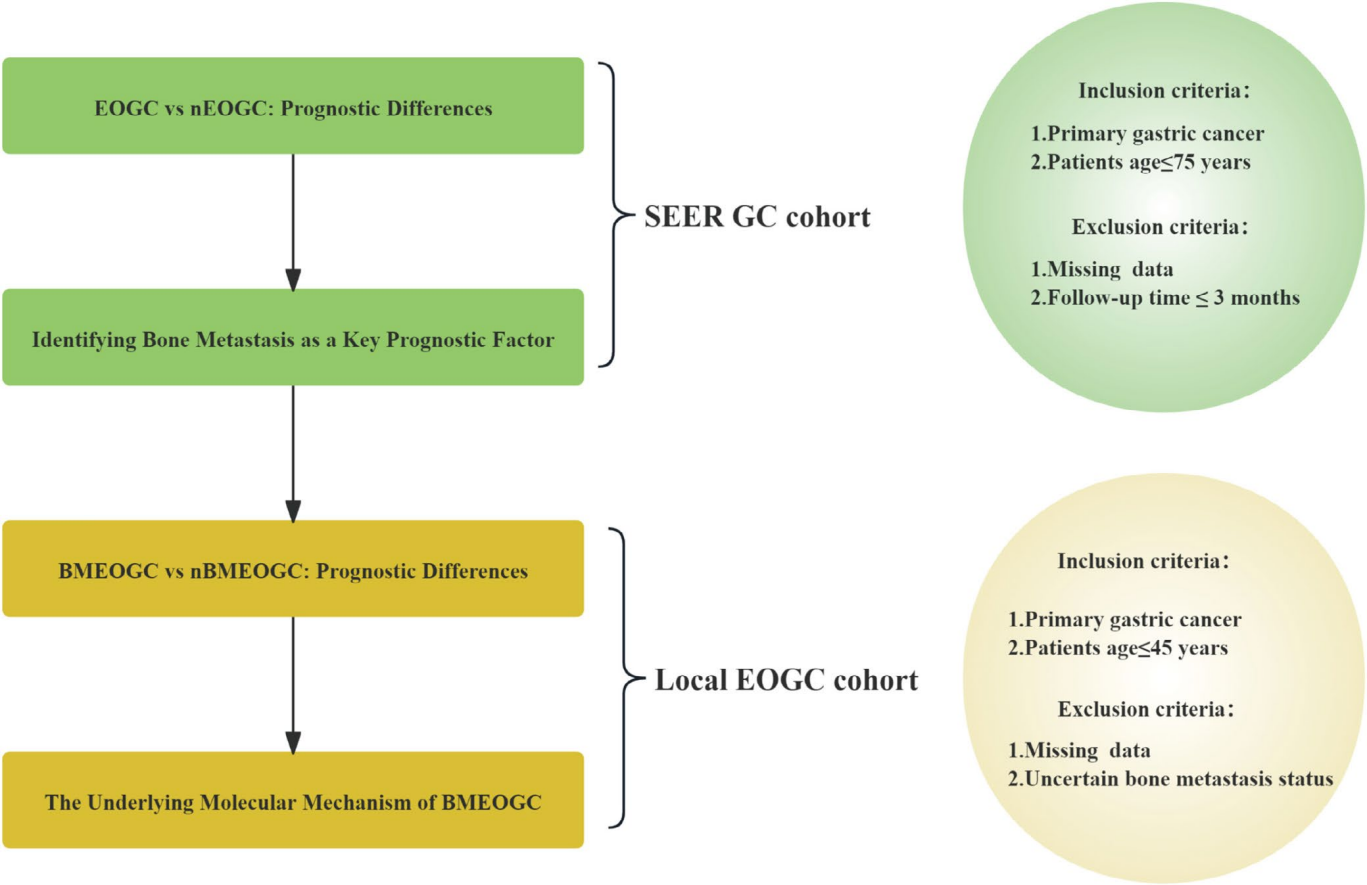
Research and design flow chart.

All data for this study were collected retrospectively, with the inclusion criteria requiring a confirmed diagnosis of primary gastric adenocarcinoma based on the American Joint Committee on Cancer (AJCC) 8th edition staging system. For the local cohort, patients aged ≤ 45 years were included, while the SEER cohort included patients aged ≤ 75 years. Moreover, both cohorts required complete clinical and pathological data. The SEER cohort specifically excluded patients with a follow‐up period of less than 3 months. The workflow of the research and design is outlined in Figure 1.

### 2.2 Cytokine Detection Using Luminex Technology

For cytokine detection, the Luminex 100/200 System was employed using a custom 34‐cytokine panel. The samples were prepared by centrifuging human plasma or serum at 3000 rpm for 10 min to collect the supernatant. The Human Standard Mix was reconstituted, vortexed, and diluted before use. The Capture Bead Mix was prepared and combined with the samples, followed by overnight incubation at 4°C. Post‐incubation, the samples were washed and incubated with Biotinylated Detection Antibody and subsequently with Streptavidin‐PE. After a final washing, the samples were read on the Luminex system. Data analysis was performed using MilliplexAnalyst software to ensure the standard curves met the quality control standards with a coefficient of variation of less than 15%.

### 2.3 Statistical Analysis

The statistical methods employed in this study were designed to ensure the validity and reliability of the results. Descriptive statistics were used to summarize the baseline clinical characteristics of the study cohorts. Categorical variables were expressed as frequencies and percentages, and comparisons between groups were conducted using the chi‐squared test or Fisher's exact test, as appropriate. Survival outcomes, including overall survival (OS) and cancer‐specific survival (CSS), were evaluated using Kaplan–Meier survival curves. The log‐rank test assessed the significance of differences between groups, with survival rates at specific time points calculated and compared. Both logistic regression and Cox proportional hazards regression models were applied to identify independent prognostic factors. Initially, univariate logistic and Cox regression analyses were performed to explore associations between clinical variables and outcomes. Variables with a *p* < 0.05 were subsequently included in multivariate regression models to determine independent predictors of survival. Results were presented as hazard ratios (HR) with 95% confidence intervals (CI). The sample size in this study was determined by the available cohort size, as is typical in retrospective analyses. The full dataset of eligible patients meeting the inclusion criteria was utilized, ensuring sufficient statistical power for subgroup analyses and multivariate modeling. Censored data were appropriately handled in survival analyses using Kaplan–Meier survival curves and Cox proportional hazards models, both of which account for right‐censored data. These approaches ensured the accurate estimation of survival probabilities and the identification of prognostic factors. Mediation analysis was conducted using the “mediation” package in R to assess the indirect effects of clinical factors on the relationship between EOGC and patient prognosis. Strict criteria were followed in selecting mediating variables, which were chosen based on their close association with the pathogenesis of EOGC and patient prognosis. The analysis was performed under the assumptions of linearity and no confounding bias, including calculations of indirect, direct, and total effects. The significance of the mediation effects was evaluated using bootstrapping methods. Additionally, the Wilcoxon test was employed to compare clinical and biomarker data between the bone metastasis early‐onset gastric cancer (BMEOGC) and non‐bone metastasis early‐onset gastric cancer (nBMEOGC) groups. All statistical analyses were conducted using R software (R Project, Version 4.1.2), with a *p* < 0.05 considered statistically significant.

## 3 Result

### 3.1 EOGC Associated With Poorer Prognosis Compared to nEOGC

In the SEER database, 8447 GC patients were included, comprising 795 EOGC patients aged ≤ 45 years and 7652 nEOGC patients (45 < age ≤ 75). Table 1 summarizes the general characteristics of these patients. Significant differences were observed between the two age groups in several aspects. The gender distribution showed a higher proportion of females in the EOGC group (45.9% vs. 30.1%, *p* < 0.01). Tumor location also varied significantly, with the EOGC group having more tumors in the middle and overlapped regions (29.7% and 11.1%, respectively), compared to the lower and upper regions in the nEOGC group (23.7% and 46.4%, respectively, *p* < 0.01). Tumor grade was predominantly poorly differentiated and undifferentiated in both groups but had a significantly higher proportion in the EOGC group (80.5% vs. 63.1%, *p* < 0.01). Regarding tumor stage, the EOGC group had a higher proportion of advanced T stages, with T3 and T4 being more common (36.4% and 31.8%, respectively) compared to the nEOGC group (39.2% and 19.6%, respectively, *p* < 0.01). Similarly, the N stage distribution indicated a higher proportion of advanced N stages (N2 and N3) in the EOGC group (17.0% and 14.3%, respectively) compared to the nEOGC group (14.6% and 9.5%, respectively, *p* < 0.01). The presence of distant metastasis was significantly higher in the EOGC group (33.1% vs. 20.4%, *p* < 0.01). Additionally, a higher percentage of EOGC patients received chemotherapy (83.9% vs. 70.9%, *p* < 0.01).

**TABLE 1:** The clinicopathological characteristics in EOGC patients of SEER cohort.

Characteristic	Overall (*N* = 8447)	nEOGC (*N* = 7652)	EOGC (*N* = 795)	*p*
Gender				
Female	2671 (31.6%)	2306 (30.1%)	365 (45.9%)	< 0.01
Male	5776 (68.4%)	5346 (69.9%)	430 (54.1%)	
Location				
Lower	2018 (23.9%)	1817 (23.7%)	201 (25.3%)	< 0.01
Middle	1933 (22.9%)	1697 (22.2%)	236 (29.7%)	
Overlapped	675 (8.0%)	587 (7.7%)	88 (11.1%)	
Upper	3821 (45.2%)	3551 (46.4%)	270 (34.0%)	
Grade				
Well	469 (5.6%)	437 (5.7%)	32 (4.0%)	< 0.01
Moderately	2508 (29.7%)	2385 (31.2%)	123 (15.5%)	
Poorly and undifferentiated	5470 (64.8%)	4830 (63.1%)	640 (80.5%)	
Tstage				
T1	2340 (27.7%)	2181 (28.5%)	159 (20.0%)	< 0.01
T2	1068 (12.6%)	974 (12.7%)	94 (11.8%)	
T3	3288 (38.9%)	2999 (39.2%)	289 (36.4%)	
T4	1751 (20.7%)	1498 (19.6%)	253 (31.8%)	
Nstage				
N0	3651 (43.2%)	3355 (43.8%)	296 (37.2%)	< 0.01
N1	2707 (32.0%)	2457 (32.1%)	250 (31.4%)	
N2	1250 (14.8%)	1115 (14.6%)	135 (17.0%)	
N3	839 (9.9%)	725 (9.5%)	114 (14.3%)	
Mstage				
M0	6626 (78.4%)	6094 (79.6%)	532 (66.9%)	< 0.01
M1	1821 (21.6%)	1558 (20.4%)	263 (33.1%)	
Stage				
I	2247 (26.6%)	2112 (27.6%)	135 (17.0%)	< 0.01
II	2491 (29.5%)	2301 (30.1%)	190 (23.9%)	
III	1888 (22.4%)	1681 (22.0%)	207 (26.0%)	
IV	1821 (21.6%)	1558 (20.4%)	263 (33.1%)	
Chemotherapy				
No/unknown	2357 (27.9%)	2229 (29.1%)	128 (16.1%)	< 0.01
Yes	6090 (72.1%)	5423 (70.9%)	667 (83.9%)	

**FIGURE 2:**
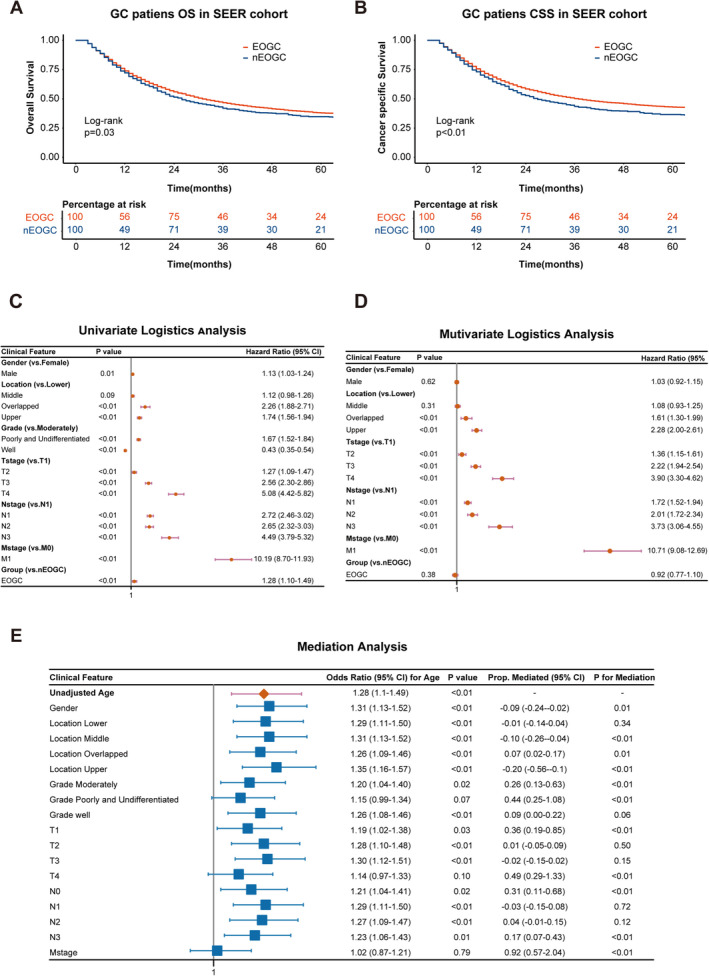
Prognostic analysis and mediation analysis of EOGC patients in the SEER cohort. Kaplan–Meier survival curves for OS and CSS in EOGC versus nEOGC patients within the SEER cohort (A, B). Univariate and multivariate logistic regression analyses of GC (C, D). Mediation analysis assessing the indirect effects of various clinical factors on the relationship between age and prognosis in EOGC patients (E).

To further understand the prognosis of EOGC patients, we performed a survival analysis using Kaplan–Meier survival curves. Regarding OS, patients in the EOGC group exhibited a 1‐year survival rate of 72.2%, a 3‐year survival rate of 42.0%, and a 5‐year survival rate of 34.8%. In contrast, patients in the nEOGC group had a 1‐year survival rate of 73.9%, a 3‐year survival rate of 46.7%, and a 5‐year survival rate of 38.0%, with significant differences between the two groups (*p* = 0.03) (Figure 2A). Similarly, for CSS, the EOGC group showed a 1‐year survival rate of 73.1%, a 3‐year survival rate of 43.6%, and a 5‐year survival rate of 36.7%, compared to 75.6%, 50.2%, and 43.0% in the nEOGC group (*p* < 0.01) (Figure 2B).

### 3.2 Bone Metastasis as a Mediating Factor in Poor Prognosis of EOGC

To further investigate the clinical factors influencing the prognosis of EOGC, we conducted univariate and multivariate statistical analyses related to survival and risk factors. In the univariate logistic regression analysis, EOGC was associated with poorer patient outcomes (HR = 1.28, *p* < 0.01) (Figure 2C). However, after adjusting for other variables in the multivariate analysis, EOGC was no longer an independent factor affecting patient prognosis (HR = 0.92, *p* = 0.38) (Figure 2D), a result consistent with the findings from the Cox regression analysis (Figure S2). Furthermore, both the logistic and Cox regression analyses revealed that tumor location, T stage, N stage, and distant metastasis are independent prognostic factors in GC patients. These results underscore the significance that age alone does not independently influence prognosis, despite the more aggressive disease typically seen in EOGC patients.

Given the potential for age impact on prognosis to be mediated by certain clinical characteristics specific to early‐onset cases, we conducted a mediation analysis to assess the intermediary roles of basic clinical information and tumor‐related characteristics (Figure 2E). The results indicated that when metastasis was considered as a mediator, the direct effect of age on prognosis was no longer significant (OR: 1.02, *p* = 0.79). The mediation analysis showed that 0.92 of the effect of age on prognosis was mediated through metastasis (*p* < 0.01), highlighting the critical role of metastasis in the prognostic pathway for EOGC patients. Furthermore, subgroup analysis of metastatic sites revealed that bone metastasis was significantly more prevalent in the EOGC group (3.6%) compared to the nEOGC group (2.0%) (*p* < 0.01) (Table 2). However, no significant differences were observed between the two groups regarding liver, lung, or brain metastasis. These findings suggest that bone metastasis is more common among younger gastric cancer patients, while the occurrence of metastasis in other organs does not differ significantly with age.

**TABLE 2:** Subgroup analysis of metastasis sites in nEOGC and EOGC patients.

Metastatic sites	nEOGC (*N* = 7652)	EOGC (*N* = 795)	*p*
Bone			
No	7498 (98.0%)	766 (96.4%)	< 0.01
Yes	154 (2.0%)	29 (3.6%)	
Liver			
No	7030 (91.9%)	736 (92.6%)	0.53
Yes	622 (8.1%)	59 (7.4%)	
Lung			
No	7459 (97.5%)	777 (97.7%)	0.75
Yes	193 (2.5%)	18 (2.3%)	
Brain			
No	7630 (99.7%)	792 (99.6%)	0.92
Yes	22 (0.3%)	3 (0.4%)	

### 3.3 Bone Metastasis as an Independent Risk Factor in EOGC Patients

Turning our attention to the local cohort, we analyzed the baseline clinical characteristics of patients with BMEOGC, as shown in Table 3. Gender distribution showed no significant difference, with females comprising 53.1% of the BMEOGC group and 52.7% of the nBMEOGC group. Furthermore, although tumor location did not significantly differ, overlapped tumors were more common in the BMEOGC group (20.4% vs. 10.4%). Both groups predominantly had poorly and undifferentiated tumors (91.8% in BMEOGC vs. 88.5% in nBMEOGC). In addition, the median BMI of the BMEOGC group was 19.53 kg/m^2^, lower than that of the nBMEOGC group (20.55 kg/m^2^; *p* = 0.03), and the BMEOGC group had a higher prevalence of advanced T and N stages (*p* < 0.01). All BMEOGC patients were in stage IV, compared to 35.4% in the nBMEOGC group (*p* < 0.01). Chemotherapy was widely used in both groups, slightly more in BMEOGC (87.8% vs. 77.2%, *p* = 0.13). Surgical intervention was significantly lower in the BMEOGC group, with only 28.6% undergoing primary tumor resection compared to 78.6% in the nBMEOGC group (*p* < 0.01). These findings highlight the advanced disease and poorer nutritional status in BMEOGC patients, underscoring the need for tailored management in this subgroup.

**TABLE 3:** The clinicopathological characteristics in BMEOGC patients of the local cohort.

Characteristic	Overall (*N* = 413)	nBMEOGC (*N* = 364)	BMEOGC (*N* = 49)	*p*
Gender				
Female	218 (52.8%)	192 (52.7%)	26 (53.1%)	1
Male	195 (47.2%)	172 (47.3%)	23 (46.9%)	
BMI	20.34 (18.36,22.68)	20.55 (18.44,22.77)	19.53 (17.56,21.93)	0.03
Location				
Lower	155 (37.5%)	142 (39.0%)	13 (26.5%)	0.13
Middle	153 (37.0%)	135 (37.1%)	18 (36.7%)	
Overlapped	48 (11.6%)	38 (10.4%)	10 (20.4%)	
Upper	57 (13.8%)	49 (13.5%)	8 (16.3%)	
Grade				
Well	7 (1.7%)	7 (1.9%)	0 (0.0%)	0.56
Moderately	39 (9.4%)	35 (9.6%)	4 (8.2%)	
Poorly and undifferentiated	367 (88.9%)	322 (88.5%)	45 (91.8%)	
Tstage				
T1–2	77 (18.6%)	77 (21.2%)	0 (0.0%)	< 0.01
T3	187 (45.3%)	163 (44.8%)	24 (49.0%)	
T4	149 (36.1%)	124 (34.1%)	25 (51.0%)	
Nstage				
N0	96 (23.2%)	95 (26.1%)	1 (2.0%)	< 0.01
N1	76 (18.4%)	71 (19.5%)	5 (10.2%)	
N2	96 (23.2%)	89 (24.5%)	7 (14.3%)	
N3	145 (35.1%)	100 (29.9%)	36 (73.5%)	
Metastasis				
Without metastasis	235 (56.9%)	235 (64.6%)	0 (0.0%)	< 0.01
Other metastasis	129 (31.2%)	129 (35.4%)	0 (0.0%)	
Bone metastasis	49 (11.9%)	0 (0.0%)	49 (100.0%)	
Stage				
I	57 (13.8%)	57 (15.7%)	0 (0.0%)	< 0.01
II	74 (17.9%)	74 (20.3%)	0 (0.0%)	
III	106 (25.7%)	106 (29.1%)	0 (0.0%)	
IV	176 (42.6%)	127 (34.9%)	49 (100.0%)	
Chemotherapy				
No	89 (21.5%)	83 (22.8%)	6 (12.2%)	0.13
Yes	324 (78.5%)	281 (77.2%)	43 (87.8%)	
Primary tumor resection				
No	113 (27.4%)	78 (21.4%)	35 (71.4%)	< 0.01
Yes	300 (72.6%)	286 (78.6%)	14 (28.6%)	

**FIGURE 3:**
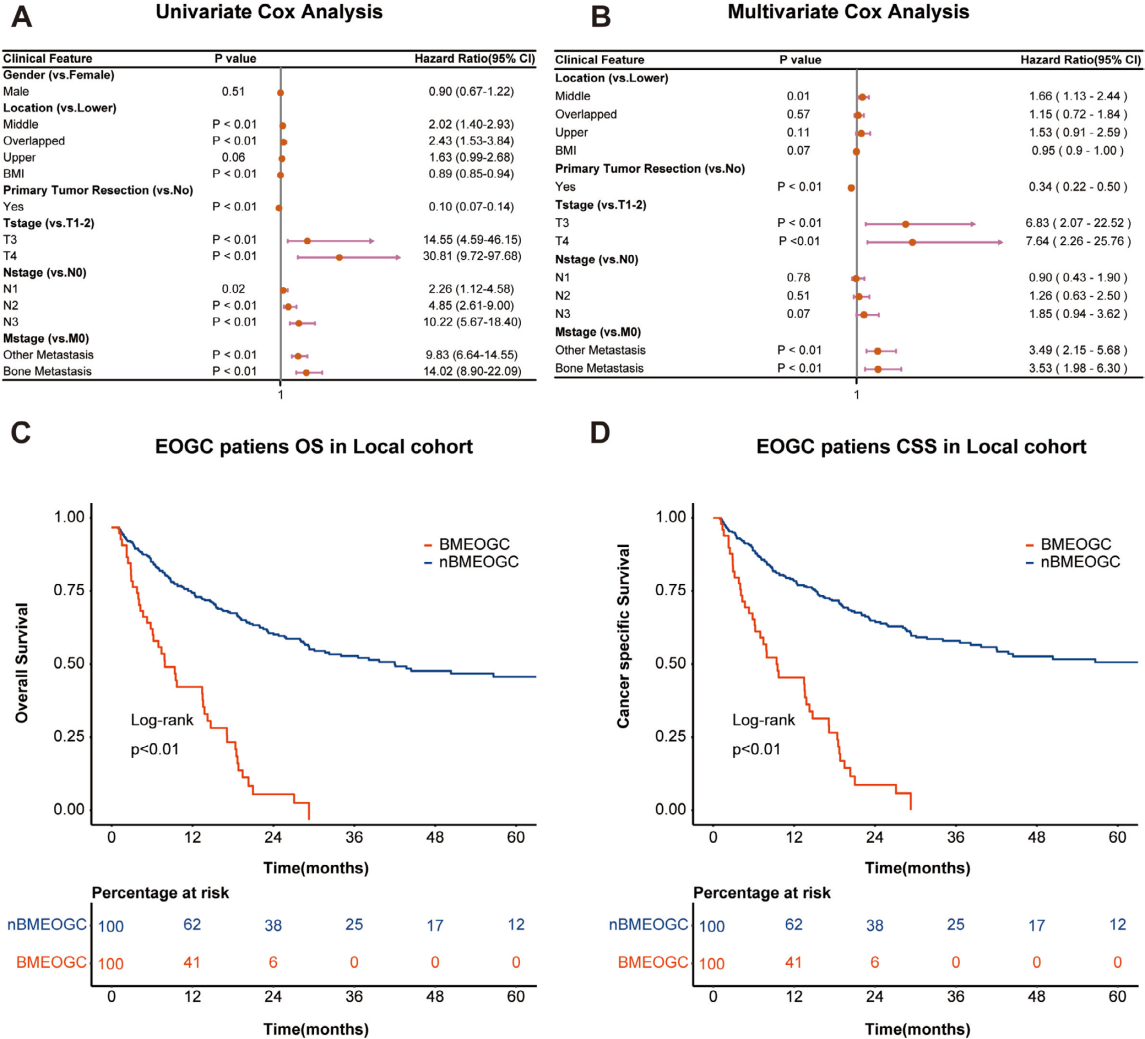
Prognostic analysis of BMEOGC patients in the local cohort. Univariate and multivariate Cox regression analyses of prognostic factors in EOGC patients (A, B). Kaplan–Meier survival curves for overall survival and cancer‐specific survival in BMEOGC versus nBMEOGC patients within the local cohort (C, D).

Using Cox proportional hazards regression, we evaluated the prognostic factors associated with EOGC. In the univariate analysis, bone metastasis was identified as a significant factor impacting prognosis (HR = 14.02, *p* < 0.01) (Figure 3A). This association persisted in the multivariate analysis, where bone metastasis (HR = 3.53, *p* < 0.01) and T stage were independent adverse prognostic factors for EOGC (Figure 3B). Survival analysis further reinforced these findings, revealing significant differences in both OS and CSS between the BMEOGC and nBMEOGC groups (Figure 3C,D). The BMEOGC group exhibited significantly poorer survival outcomes compared to the nBMEOGC group, highlighting the more aggressive clinical course of BMEOGC. These results emphasize the necessity for tailored therapeutic approaches to improve survival outcomes in this particularly high‐risk patient population.

### 3.4 BMEOGC: Immune, Inflammatory, and Coagulation Dysregulation

**FIGURE 4:**
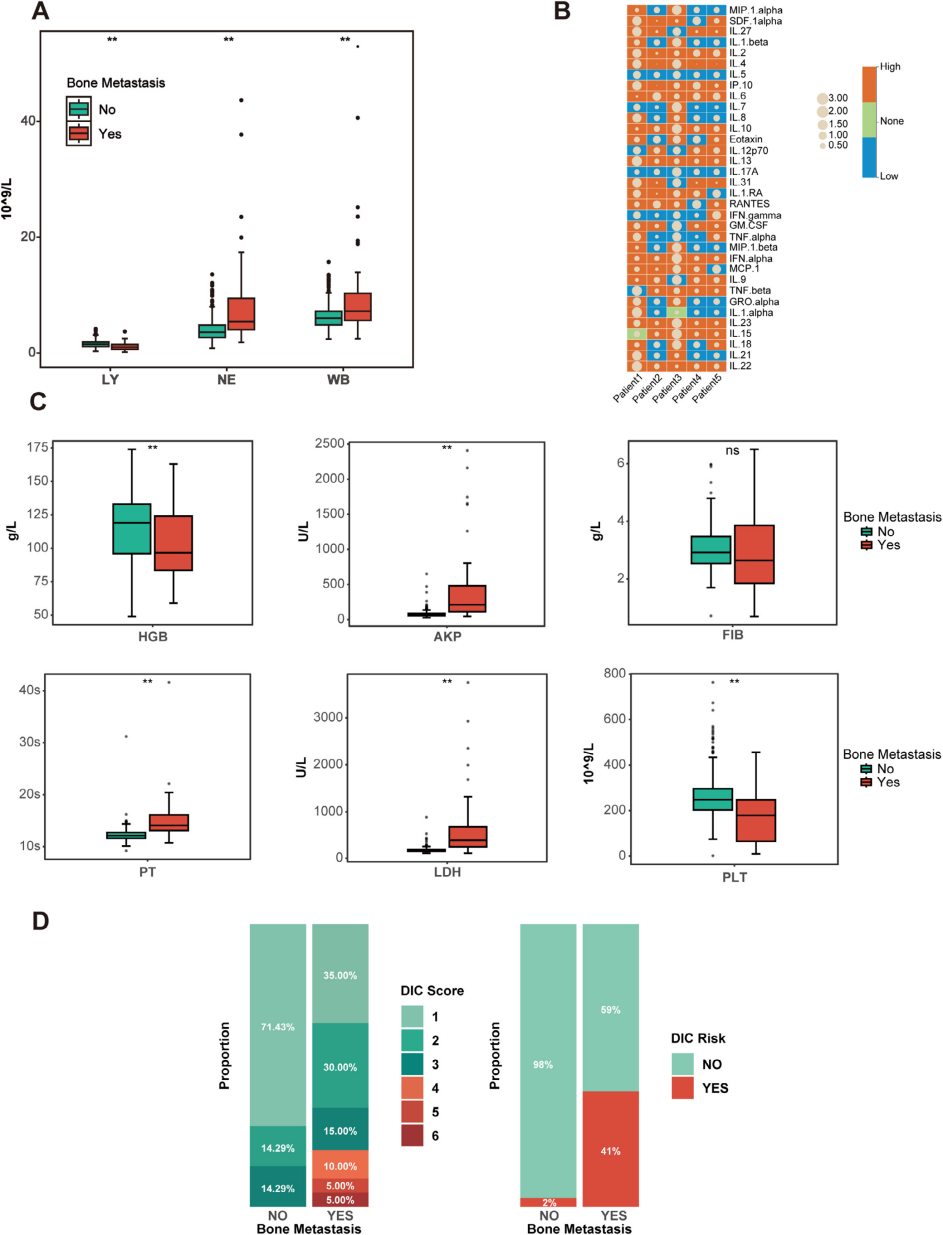
Analysis of clinical and biomarker features in BMEOGC patients. Blood cell analysis comparing lymphocyte (LY), neutrophil (NE), and white blood cell (WBC) counts between BMEOGC patients and nBMEOGC (A). Cytokine analysis showing the differential expression of cytokines in BMEOGC patients (B). Expression levels of coagulation and physiological markers in BMEOGC patients (C). DIC risk assessment in BMEOGC patients (D). “*” indicates *p* < 0.05; “**” indicates *p* < 0.01 for statistical significance.

We performed a comprehensive multidimensional biomarker analysis to further elucidate the biological mechanisms underlying the poor prognosis observed in BMEOGC patients. The blood cell analysis revealed that BMEOGC patients exhibited significantly elevated neutrophil (NE) and white blood cell (WBC) counts, coupled with a reduction in lymphocyte (LY) levels (Figure 4A). Additionally, cytokine profiling in the BMEOGC group revealed substantial elevations in various pro‐inflammatory cytokines, notably including interleukins IL‐1β, IL‐6, and IL‐8, as well as TNF‐α and GM‐CSF. This indicates an extensive inflammatory response within BMEOGC (Figure 4B). Further analysis of coagulation parameters and disseminated intravascular coagulation (DIC) scores demonstrated that BMEOGC patients exhibited significantly higher DIC scores, indicating a heightened risk of coagulopathy (Figure 4D). Concurrently, key coagulation markers, including fibrinogen (FIB) and prothrombin time (PT), showed elevated levels, while platelet count (PLT) exhibited a notable decrease in the BMEOGC group, indicating a hypercoagulable state (Figure 4C). Additionally, other related factors, such as alkaline phosphatase (AKP) and lactate dehydrogenase (LDH), were significantly elevated in the BMEOGC group. These findings emphasize the intricate interplay among inflammation, immune response, and coagulation abnormalities within the BMEOGC group, which potentially contributes to their adverse clinical outcomes.

## 4 Discussion

Recent data indicate a decline in overall GC incidence, yet EOGC is paradoxically on the rise, raising significant concerns globally [19]. This trend hampers socioeconomic development and imposes heavy financial burdens on affected families [20]. While previous studies have characterized EOGC broadly, the analysis of its prognostic factors remains contentious. Thus, this study aims to delve deeper into the prognostic factors of EOGC and offer valuable guidance for treatment.

This study employs an age‐stratified analysis to elucidate clinicopathological and prognostic differences between EOGC and nEOGC cohorts. Contrary to previous studies that observed no distinct prognostic disparities in EOGC, our results indicate worse outcomes for EOGC patients, potentially due to excluding elderly patients from comparisons [21, 22]. This finding highlights the crucial need to account for age‐specific subgroups in prognosis evaluations to minimize confounding influences.

Besides, the univariate analysis highlights that EOGC significantly impacts prognosis, aligning with previous findings that younger patients often exhibit more aggressive disease and worse survival outcomes [5, 23]. However, after adjusting for potential confounders in multivariate analyses, EOGC was not an independent prognostic factor. While EOGC is associated with adverse outcomes, its influence is likely mediated by other factors, not age. Studies have demonstrated that the highly aggressive biological behavior of EOGC, which is characterized by poor tumor differentiation, diffuse histological types, and higher rates of lymph node involvement and distant metastasis, is a crucial confounder that relates closely to poor prognosis [22, 24, 25]. These findings emphasize the importance of considering a wider clinical context and the interactions among various factors when assessing prognosis.

To further explore these interactions, a mediation analysis was conducted, revealing that metastasis mediates the relationship between age and prognosis in EOGC patients. This finding highlights the indirect role that metastasis plays in influencing the outcomes of younger GC patients. Subsequent subgroup analyses identified a particularly strong association between bone metastasis and EOGC, suggesting that bone metastasis is a critical driver of poor prognosis in this patient population. Studies have found that bone metastasis significantly disrupts physiological bone remodeling and homeostasis, leading to severe clinical complications and increased mortality rates. Unlike other metastases (such as liver, lung, or brain metastases), bone metastasis is typically characterized by osteolytic and osteogenic reactions, promoting the formation of the tumor microenvironment [26, 27, 28]. Therefore, although previous studies have recognized the general significance of metastasis in GC prognosis, our research emphasizes the unique impact of bone metastasis within the context of EOGC, revealing its pivotal role in worsening disease outcomes.

Based on these insights, this study further investigates the unique prognostic features of BMEOGC. Consistent with expectations, survival analysis demonstrated significantly poorer outcomes for patients with bone metastasis compared to those without. Both univariate and multivariate analyses robustly confirmed that bone metastasis is an independent prognostic factor in EOGC, underscoring its critical role in exacerbating adverse outcomes.

To further elucidate the mechanisms underlying the poor prognosis in BMEOGC patients, we conducted a multidimensional biomarker analysis. This revealed that BMEOGC is characterized by immunosuppression, chronic inflammation, and coagulation abnormalities. Specifically, LY counts reflect impaired immune surveillance, while elevated NE levels are associated with systemic inflammation and the secretion of pro‐tumor cytokines such as IL‐6 and TNF‐α. This imbalance, reflected by an increased neutrophil‐to‐lymphocyte ratio, has been shown in other cancers to correlate with worse prognosis and enhanced metastatic potential, including in bone metastasis [29, 30]. Studies have shown that cytokines such as IL‐6 and TNF‐α play key roles in the tumor microenvironment in the context of elevated cytokines and bone metastasis progression. IL‐6 promotes tumor cell proliferation, survival, and migration by activating the STAT3 signaling pathway [31]. In bone metastases, tumor‐derived IL‐6 stimulates osteoclast activity, increasing bone resorption and creating a favorable environment for tumor cell colonization and growth. TNF‐α influences tumor cell invasion by inducing apoptosis and inflammatory responses. Moreover, TNF‐α regulates vascular endothelial growth factor expression, facilitating tumor angiogenesis and supporting the progression of bone metastases [32]. The hypercoagulable state observed in BMEOGC patients further contributes to bone metastasis progression. Tumor cells release tissue factors that activate the coagulation system, leading to hypercoagulability. The resulting fibrin clots not only provide a scaffold for tumor cell adhesion and migration but also release growth factors such as platelet‐derived growth factor, which promotes tumor cell growth and metastasis [33]. In terms of the interaction between immune profiles and the bone microenvironment, lymphocytes and neutrophils play critical roles in tumor immune surveillance and regulation of the tumor microenvironment. T cells within the lymphocyte population can recognize and eliminate tumor cells; however, tumor cells often evade immune surveillance by secreting immunosuppressive factors such as TGF‐β, which inhibits T‐cell activity [34]. Neutrophils, while exhibiting anti‐tumor effects in early stages through phagocytosis and the release of antimicrobial substances, can be recruited by tumor cells in later stages. These tumor‐associated neutrophils release matrix metalloproteinases and other factors that enhance tumor invasion and metastasis [35]. In the bone microenvironment, interactions between immune cells, tumor cells, osteoclasts, and osteoblasts further shape the metastatic process, highlighting the complexity of immune regulation in bone metastases. Moreover, the chronic inflammatory state observed in BMEOGC patients plays a significant role in advancing tumorigenesis. Chronic inflammation is a well‐established driver of cancer progression, frequently associated with sustained activation of pro‐inflammatory pathways that support cellular proliferation, angiogenesis, and resistance to apoptosis. This inflammatory milieu not only facilitates the initiation of metastasis but also perpetuates its expansion, thereby worsening the prognosis for BMEOGC patients [36, 37, 38]. More than these factors, coagulation abnormalities, particularly hypercoagulability, manifest as a crucial aspect of BMEOGC. This hypercoagulable state supports the survival of circulating tumor cells by providing a scaffold for their adhesion and colonization at distant metastatic sites, which is essential for the establishment of bone metastases. The hypercoagulable state also increases the risk of thromboembolic events, further complicating clinical management and contributing to the overall poor prognosis of BMEOGC patients [39, 40, 41]. The interaction between coagulation disorders and metastatic progression highlights the necessity for integrated therapeutic approaches.

Overall, this study provides a detailed understanding of bone metastasis in EOGC, offering novel insights that have been insufficiently explored in previous research. Firstly, it uniquely identifies bone metastasis as a mediator of poor prognosis in EOGC, a pivotal insight that opens new avenues for therapeutic strategies. Secondly, leveraging multicenter cohort data and multidimensional analysis, this research elucidates the underlying reasons for poor prognosis in BMEOGC from the perspectives of inflammation, immune response, and coagulation balance, setting the stage for targeted interventions. Despite its strengths, this study faces several limitations. The retrospective design may introduce selection bias, potentially affecting the generalizability of the findings. While focusing on clinical and biomarker data, the study does not include genetic and molecular analyses, such as TP53, HER2, or microsatellite instability, which are critical in gastric cancer progression and metastasis. Incorporating these markers in future research could provide deeper insights into EOGC prognosis. Additionally, the cut‐off values for abnormal biomarkers, derived from literature and clinical guidelines, may vary across laboratories and populations, potentially impacting reproducibility. Refining and standardizing these thresholds will be essential to improve their applicability. Finally, the study highlights differences in cytokine levels and immune cell populations between BMEOGC and nBMEOGC but does not explore the upstream regulators and downstream effectors driving these changes. Future studies should integrate transcriptomic and proteomic analyses to uncover key pathways and develop targeted therapies for EOGC.

## 5 Conclusion

In conclusion, our research establishes that EOGC patients have poorer prognoses, with bone metastasis mediating this adverse outcome. Through multicenter cohort analysis, it was demonstrated that BMEOGC is strongly associated with immune suppression, chronic inflammation, and coagulation imbalances. This highlights the need for targeted therapeutic strategies.

## Author Contributions

Shi Yin, Caiqin Wang, Huashe Wang, and Ziying Zhang contributed to the conception and design of the study. Xiaohui Zhai was responsible for data collection. Shi Yin and Yaoying Li performed the data analysis and interpretation. Ruixin Zeng provided critical revisions to the manuscript for important intellectual content. Shi Yin, Di Zhang, and Xiaoqing Sun supervised the study and contributed to the drafting of the manuscript. All authors reviewed and approved the final manuscript.

## Ethics Statement

This retrospective cohort study was conducted in accordance with the Declaration of Helsinki and received approval from the Clinical Research Ethics Committee of the Sixth Affiliated Hospital of Sun Yat‐sen University (No. 2021ZSLYEC‐325). The need for written informed consent was waived by the Clinical Research Ethics Committee due to the retrospective nature of this study and the use of de‐identified patient data. Data from the SEER database cohort were accessed and analyzed in full compliance with the strict requirements and terms of SEER's data use agreement.

## Consent

The authors have nothing to report.

## Conflicts of Interest

The authors declare no conflicts of interest.

## Supporting information


**Figure S1.** The flowchart depicts the selection process for the study cohorts.

**Figure S2.** Univariate and multivariate Cox regression analyses of prognostic factors in GC patients (A, B).

Data S1.

## Data Availability

The data supporting the findings of this study are available from the corresponding author upon reasonable request. The datasets generated and/or analyzed during the current study are not publicly available due to privacy concerns but are available from the corresponding author, Caiqin Wang, upon reasonable request.
