# Supplementary figures and images for: Bone Metastasis Mediates Poor Prognosis in Early‐Onset Gastric Cancer: Insights Into Immune Suppression, Coagulopathy, and Inflammation
Source: Cancer Med. 2025 Mar 5;14(5):e70737. doi: 10.1002/cam4.70737 (PMC11880774; doi:10.1002/cam4.70737)

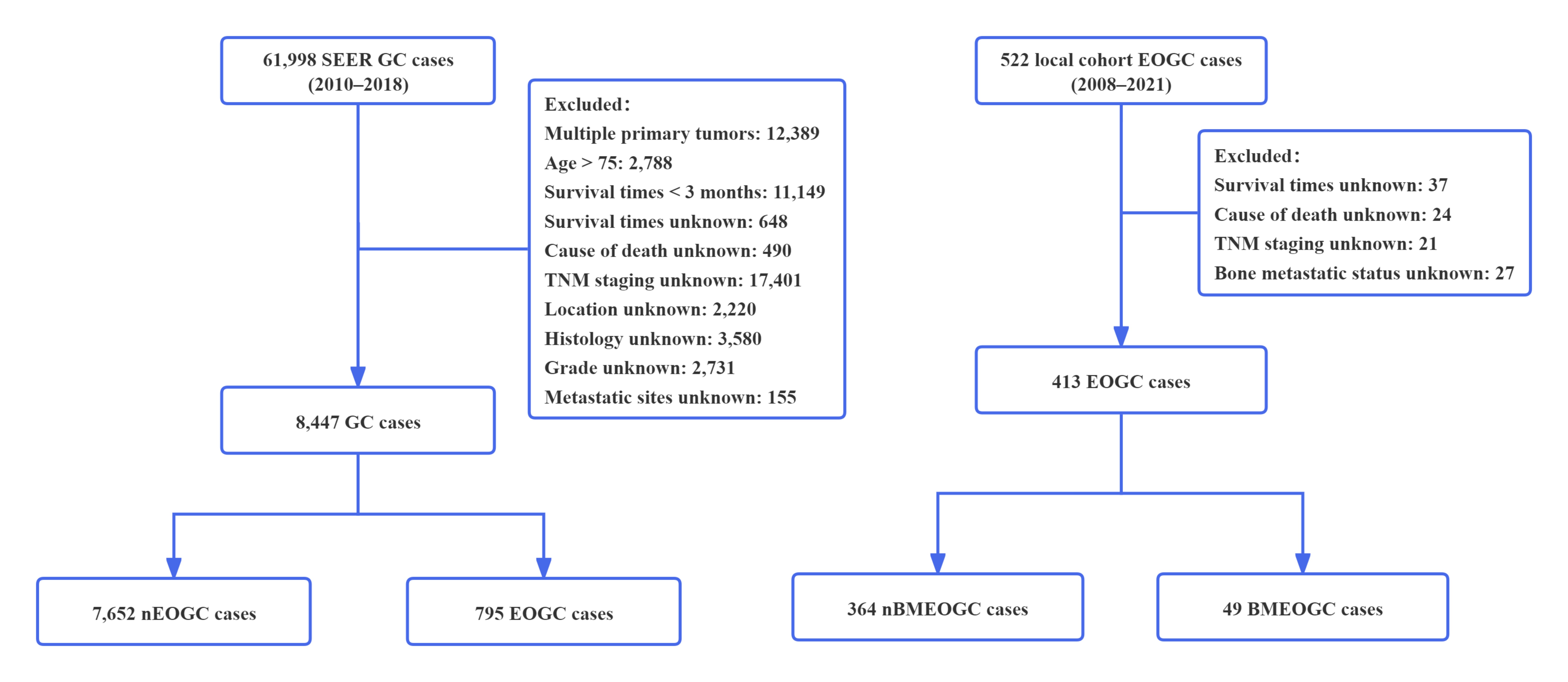

Supplement: Supplementary file 1 — Figure S1. The flowchart depicts the selection process for the study cohorts. [file CAM4-14-e70737-s001.tif]

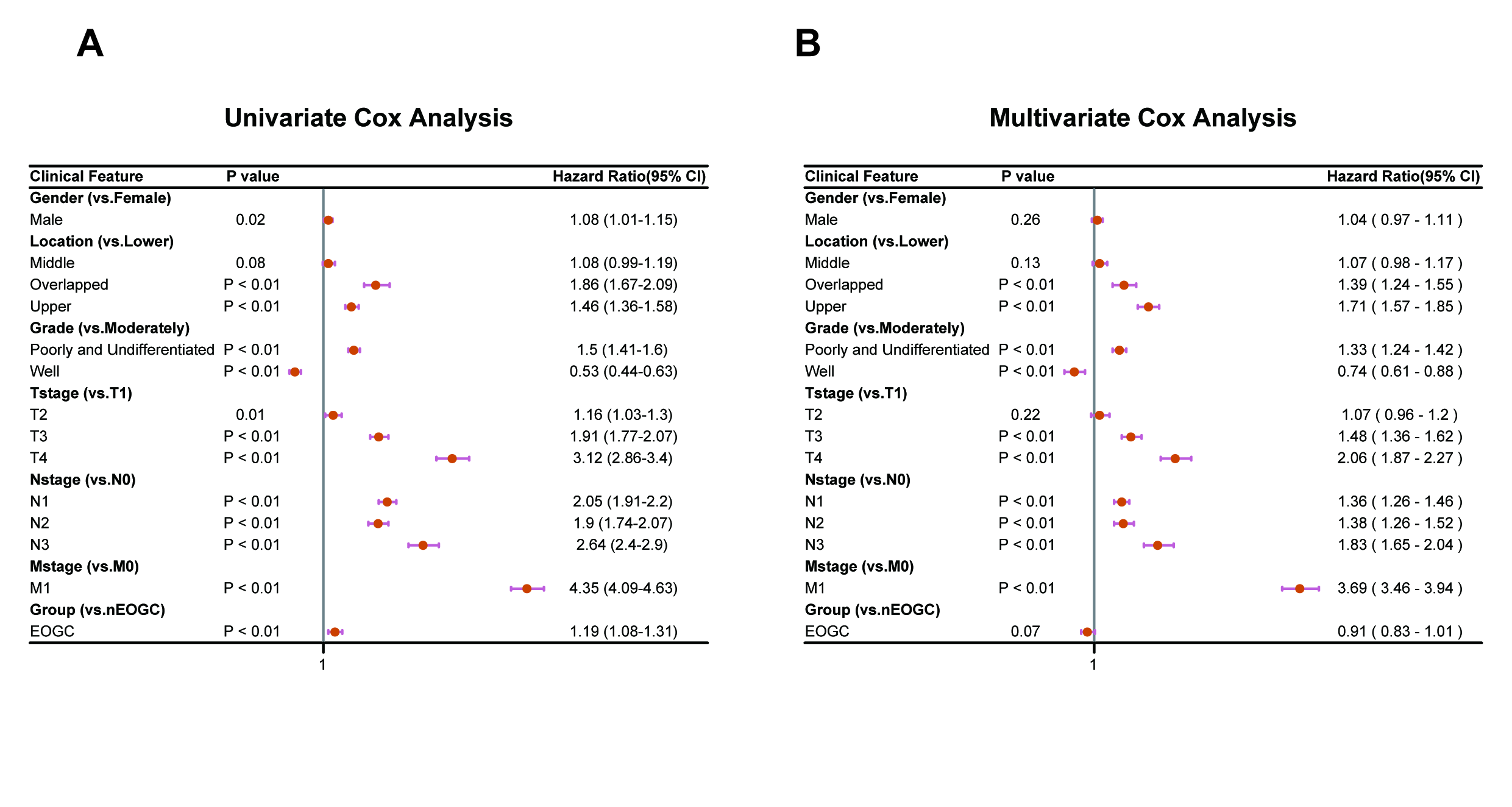

Supplement: Supplementary file 2 — Figure S2. Univariate and multivariate Cox regression analyses of prognostic factors in GC patients (A, B). [file CAM4-14-e70737-s002.tif]
