# Supplementary material for: Bone Metastasis Mediates Poor Prognosis in Early‐Onset Gastric Cancer: Insights Into Immune Suppression, Coagulopathy, and Inflammation
Source: Cancer Med. 2025 Mar 5;14(5):e70737. doi: 10.1002/cam4.70737 (PMC11880774; doi:10.1002/cam4.70737)

Supplementary Information

# Title: Bone Metastasis Mediates Poor Prognosis in Early-Onset Gastric Cancer: Insights into Immune Suppression, Coagulopathy, and Inflammation

# Running Head: Bone Metastasis in Early-Onset Gastric Cancer

Shi Yin^1†^, Xiaohui Zhai^2,3,4†^, Yaoying Li^5†^, Ruixin Zeng^6^, Di Zhang^7^, Xiaoqing Sun^8^, Ziying Zhang^9^, Huashe Wang^10*^, Caiqin Wang^11*^

^1^The Affiliated LiHuiLi Hospital of Ningbo University

^2^Department of Medical Oncology, the Sixth Affiliated Hospital of Sun-Yat Sen University, Guangzhou, 510655, P.R. China

^3^Guangdong Provincial Key Laboratory of Colorectal and Pelvic Floor Diseases, the Sixth Affiliated Hospital, Sun Yat-sen University

^4^Biomedical Innovation Center, the Sixth Affiliated Hospital, Sun Yat-sen University

^5^State Key Laboratory of Oncology in South China, Sun Yat-sen University Cancer Center, Guangzhou, China

^6^Department of Medical Oncology, State Key Laboratory of Oncology in South China, Guangdong Provincial Clinical Research Center for Cancer, Sun Yat-sen University Cancer Center, Guangzhou 510060, P. R. China

^7^Ningbo Geriatric Rehabilitation Hospital

^8^Department of Intensive Care Medicine (ICU), State Key Laboratory of Oncology in South China, Guangdong Provincial Clinical Research Center for Cancer, Sun Yat-sen University Cancer Center, Guangzhou 510060, P.R.China

^9^Department of Radiation Oncology, Hunan Cancer Hospital and the Affiliated Cancer Hospital of Xiangya School of Medicine, Central South University, Changsha, Hunan, P.R. China

^10^Department of Gastrointestinal Surgery, the Six Affiliated Hospital, Sun Yat-sen University, Guangzhou 510655, China

^11^Department of Lymphoma and Hematology, the Affiliated Cancer Hospital of Xiangya School of Medicine, Central South University, Hunan Cancer Hospital, Changsha 410013, Hunan, China

^†^These authors contributed equally.

Correspondence: Huashe Wang, Email: wanghsh@mail.sysu.edu.cn;

Caiqin Wang, Email: wangcaiqin@hnca.org.cn.

**Figure S1.** The flowchart depicts the selection process for the study cohorts.

**
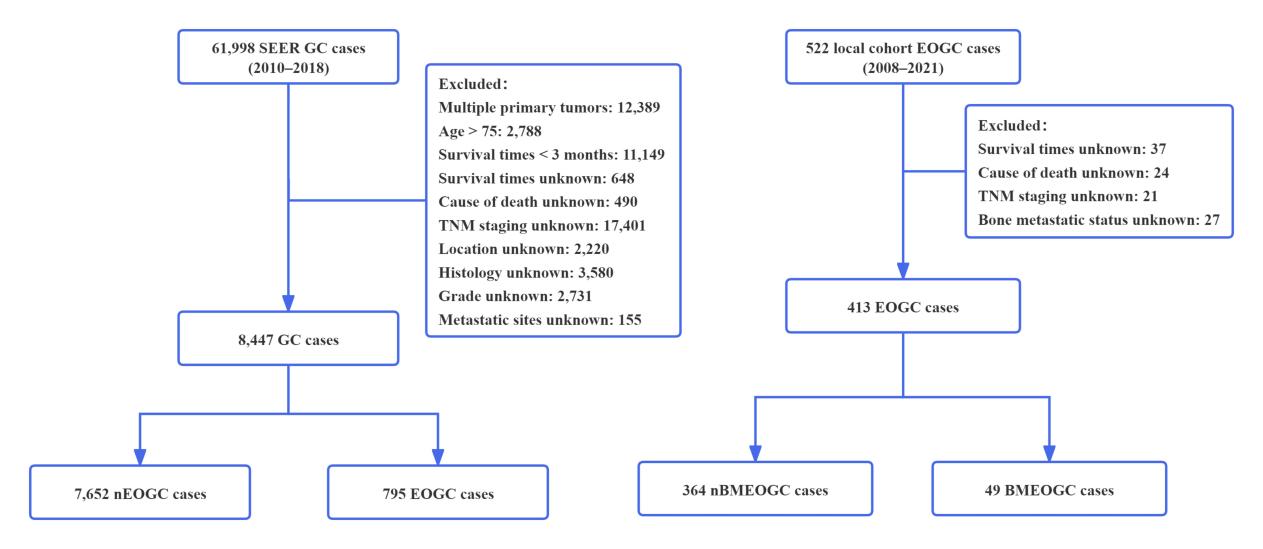
**

**Figure S2.** Univariate and multivariate Cox regression analyses of prognostic factors in GC patients (A, B).


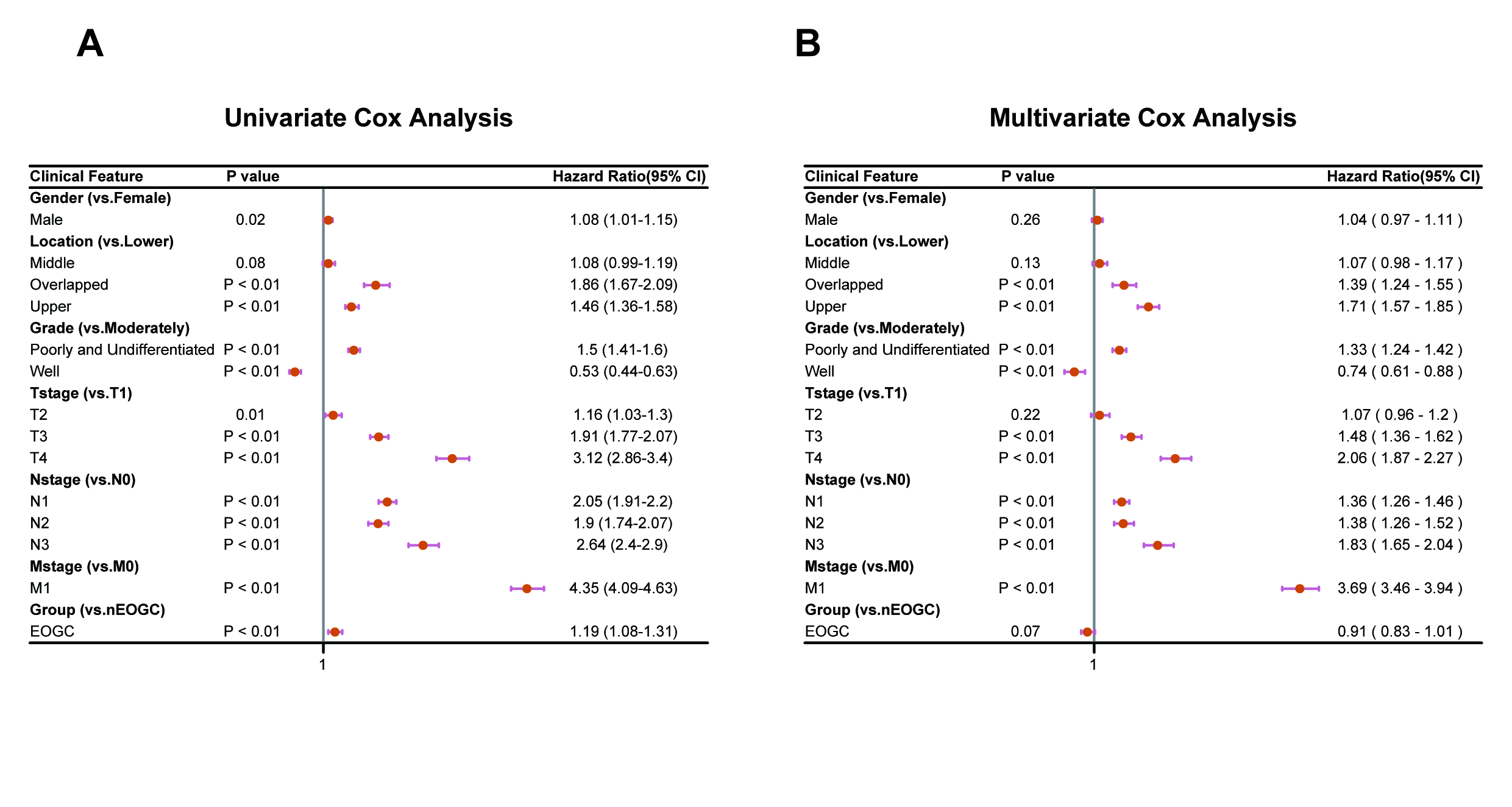

Supplement: Supplementary file 3 — Data S1. [file CAM4-14-e70737-s003.docx]
